# Choledochal Cysts in Children: A Single-Center Study in South India

**DOI:** 10.7759/cureus.86499

**Published:** 2025-06-21

**Authors:** Venkat Kumar Raju Cherukuri, Shilpa Radhakrishnan, R. Bhanu Vikraman Pillai

**Affiliations:** 1 Pediatrics, Amrita Institute of Medical Sciences and Research Center, Kochi, IND; 2 Radiodiagnosis, Amrita Institute of Medical Sciences and Research Center, Kochi, IND; 3 Pediatric Gastroenterology, Amrita Institute of Medical Sciences and Research Center, Kochi, IND

**Keywords:** anomalous pancreaticobiliary junction, carolis disease, choledochal cysts, chronic liver disease, neonatal cholestasis

## Abstract

Objectives

Choledochal cysts are rare congenital anomalies of the biliary tract with limited data on their clinical spectrum and outcomes in pediatric populations, particularly in South India. This study aimed to analyze the clinical presentation, types, associated comorbidities, management, and long-term outcomes of choledochal cysts in children over a 20-year period at a single tertiary care center.

Methods

This retrospective audit of hospital records study was conducted at the Amrita Institute of Medical Sciences and Research Center, Kochi, Kerala, India. Data from 147 pediatric patients with choledochal cysts were analyzed, including clinical presentation, radiological findings, laboratory investigations, management, and outcomes.

Results

Among 147 patients, 103 were female (M:F ratio = 1:2.4). The mean age of the patients at presentation was 3.4 months. Abdominal pain (58%, n=85) was the most common symptom, while the classic triad of pain, jaundice, and palpable mass was rare (4%, n=6). Type I cysts (63%, n = 93) were the most frequent, followed by type IV (31%, n = 46) and Caroli’s disease (5.4%, n=8). Anomalous pancreaticobiliary junction (APBJ) was seen in 14% (n=21) of patients, of whom 43% had pancreatitis. Overall, pancreatitis occurred in 20% of patients (n=29), and renal anomalies were noted in 12% (n=18). Surgical intervention was performed in 75% (n=110) of the patients, with a mean age at surgery of 4.1 years. Histopathology revealed metaplastic changes in 3% of the cases (n=3). Long-term complications included chronic liver disease (7%, n=11) and portal hypertension (4%, n=6).

Conclusion

This study highlights the clinical profile of pediatric choledochal cysts in South India, emphasizing early diagnosis and surgical intervention, which may reduce complications and improve outcomes. Multicenter studies are needed to improve understanding of disease progression and outcomes.

## Introduction

Choledochal cysts (CCs) are rare congenital anomalies of the biliary tract, characterized by cystic dilatation of the intrahepatic and/or extrahepatic bile ducts. First described in 1723 by Vater and Ezler, CCs occur more frequently in females than in males, with a reported female-to-male ratio of 4:1 [1], and have a higher incidence in Asian populations.

Although the exact aetiology is not fully understood, Babbitt proposed that an anomalous pancreaticobiliary junction (APBJ) may play a central role [2,3]. This anomaly permits the reflux of pancreatic enzymes into the common bile duct, leading to inflammation and cystic dilatation. In 1959, Alonso-Lej et al. proposed the initial classification system for CCs, identifying four types [4], which were later expanded by Todani et al. in 1977 to include a Type V, or Caroli disease, which involves intrahepatic bile duct dilatation and may be associated with congenital hepatic fibrosis and recurrent cholangitis, and can progressively lead to portal hypertension and chronic liver disease [5].

Approximately 80% of CCs are diagnosed during childhood [6]. The classic triad of jaundice, abdominal pain, and a right upper quadrant mass is rare and seen predominantly in pediatric patients. Clinical presentations range from asymptomatic cases to life-threatening complications such as cholangitis and pancreatitis. Infants often present atypically, including with neonatal cholestasis or, less commonly, duodenal obstruction and perforation [7]. Although uncommon in children, malignant transformation has been reported, most often as cholangiocarcinoma and occasionally as gallbladder or bile duct adenocarcinoma [8]. Definitive management typically involves cyst excision with biliary-enteric reconstruction, most often via Roux-en-Y hepaticojejunostomy, to minimize long-term complications [1].

Data on CC from South India remain limited. This study will analyze the clinical spectrum, cyst types, associated comorbidities, management strategies, and long-term outcomes of pediatric CCs over 20 years at a single tertiary care center in South India.

## Materials and methods

This study was a retrospective, hospital-based clinical audit and cohort study conducted at the Department of Pediatrics and Pediatric Gastroenterology, Amrita Institute of Medical Sciences, Kochi, India. The study was approved by the Ethics Committee of Amrita School of Medicine (Approval No: ECASM-AIMS-2024-380). Data were collected over a 20-year period, from September 2004 to September 2024.

Study population

Pediatric patients (<18 years) with confirmed diagnoses of CC based on radiological and/or intraoperative findings were included. Patients with incomplete or insufficient data were excluded.

Sample size

A total of 151 cases were identified, and 147 patients were included in the final analysis after excluding four with inadequate data. The insufficiency stemmed from missing or incomplete documentation of clinical, radiological, or surgical information in the hospital records.

Data collection

Data were collected from electronic medical records, including demographic details, clinical presentation, laboratory investigations, imaging studies, surgical procedures, histopathological findings, and follow-up outcomes.

Imaging and diagnostic workup

Ultrasound was performed in all patients using high-resolution pediatric probes by experienced pediatric radiologists. Evaluations were done for cyst morphology, liver echotexture, gallbladder, and intrahepatic ductal dilatation. Magnetic resonance cholangiopancreatography (MRCP) was indicated for detailed biliary anatomy and cyst classification. It was performed in 77% of patients using a 1.5T scanner, with T2-weighted heavily MRCP sequences and contrast sequences in select patients. Endoscopic retrograde cholangiopancreatography (ERCP) was performed in 25% of patients, primarily for those with clinical or imaging evidence of pancreatitis or choledocholithiasis. Therapeutic maneuvers included stone extraction and stenting.

Classification

**Figure 1:**
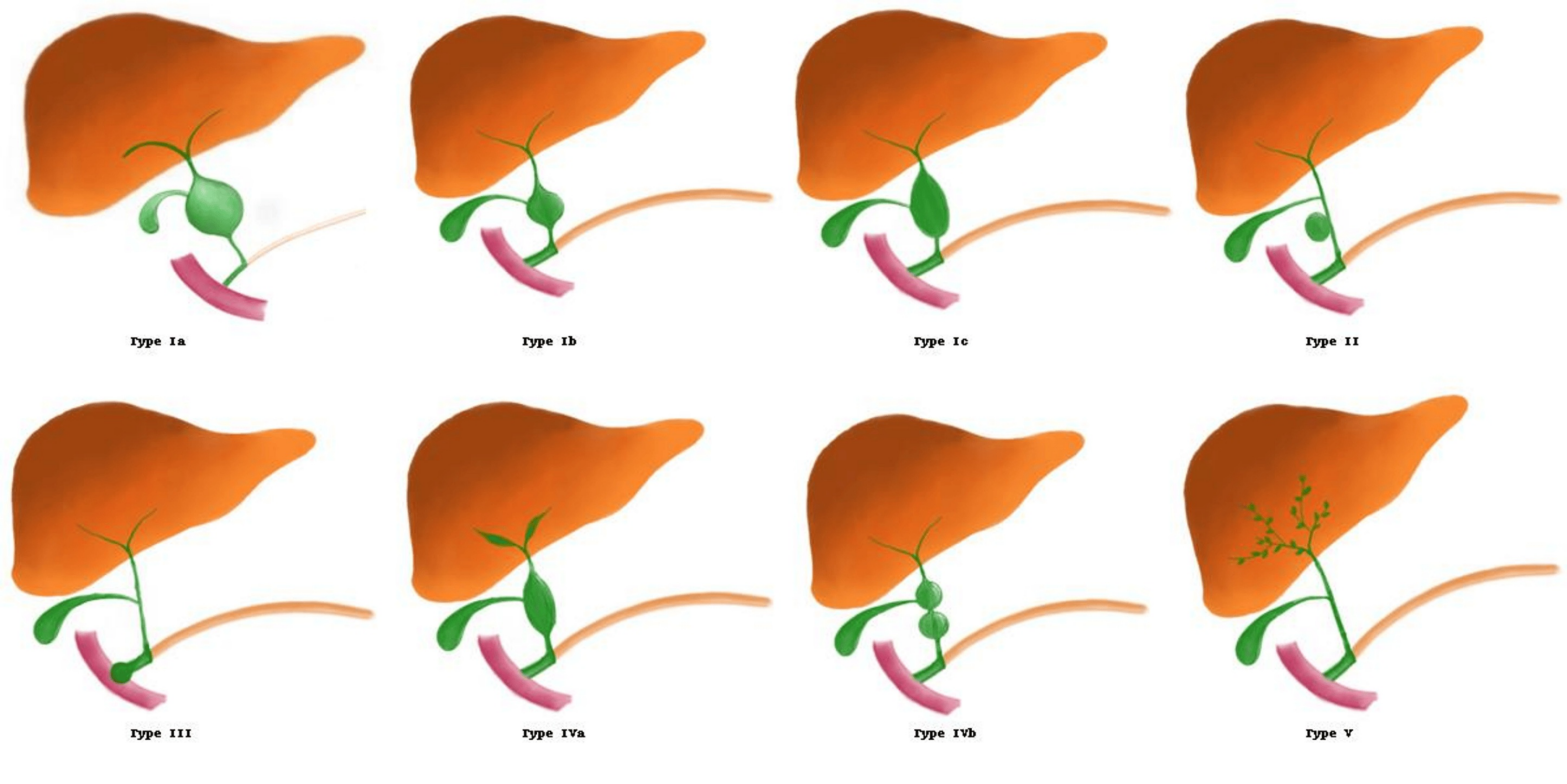
Schematic representation of Todani classification of choledochal cysts Type Ia: Cystic dilatation of the entire extrahepatic bile duct; Type Ib: Segmental (focal) dilatation of the extrahepatic bile duct; Type Ic: Fusiform dilatation of the extrahepatic bile duct; Type II: True diverticulum of the common bile duct; Type III: Choledochocele—dilatation of the intraduodenal portion of the CBD; Type IVa: Multiple cysts involving both intrahepatic and extrahepatic ducts; Type IVb: Multiple cysts limited to the extrahepatic duct; Type V: Caroli’s disease: intrahepatic ductal dilatation only Image Credit: Original artwork by Dr. Nihitha Koneru; Used with permission

Choledochal cysts were categorized using the Todani classification system [5]. A summary of the Todani classification is provided in Table 1 and Figure 1.

**Table 1:** Todani classification of choledochal cysts CBD: common bile duct

Type	Description	Subtypes
Type I	Fusiform dilatation of the extrahepatic bile duct	Ia – Cystic, Ib – Segmental, Ic – Fusiform
Type II	Diverticulum protruding from the common bile duct	–
Type III (Choledochocele)	Cystic dilatation of the distal CBD within the duodenal wall	–
Type IV	Multiple cysts involving intra- and/or extrahepatic ducts	IVa – Intra + Extra, IVb – Extra only
Type V (Caroli’s Disease)	Intrahepatic cystic dilatation only	–

Management and follow-up

Surgical intervention was performed based on individual patient characteristics. The surgical decision was made by a joint pediatric surgery-hepatology team. Roux-en-Y hepaticojejunostomy was the preferred approach. Intraoperative cholangiography was performed in select cases to delineate intrahepatic involvement. Gallbladder and cyst excision was standard; in Caroli disease, surgery was individualized based on the extent of hepatic involvement.

Histopathological analysis of excised specimens was conducted to identify inflammatory and metaplastic changes. Postoperative follow-up included outpatient visits, imaging, and liver function monitoring to detect long-term complications such as chronic liver disease and portal hypertension.

**Figure 2:**
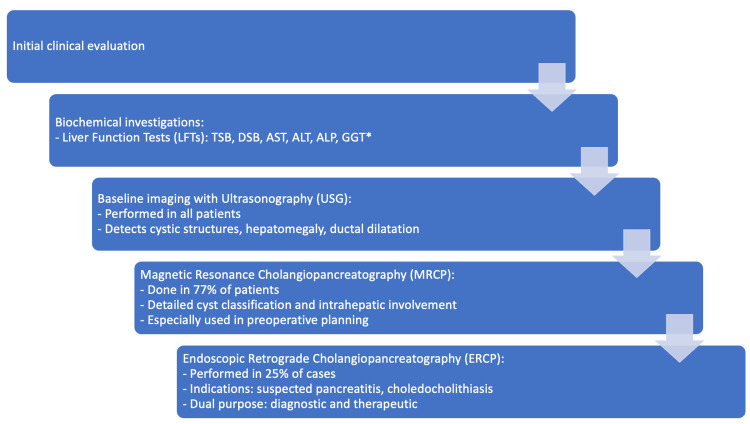
Flowchart showing the diagnostic workup of pediatric choledochal cysts *GGT testing was not performed in all cases, as data availability varied in this retrospective audit TSB: total serum bilirubin; DSB: direct serum bilirubin; AST: aspartate aminotransferase; ALT: alanine aminotransferase; GGT: gamma-glutamyl transpeptidase

Figure 2 shows the diagnostic workup followed in the cases included in the study.

Outcome definitions

Chronic Liver Disease (CLD) was defined as persistent hepatomegaly, deranged liver function tests (LFTs) beyond six months post surgery, and imaging evidence of liver fibrosis or nodularity. Portal hypertension was defined by the presence of splenomegaly, varices on imaging or endoscopy, or thrombocytopenia with splenomegaly. Pancreatitis was diagnosed based on the INSPPIRE (International Study group of Pediatric Pancreatitis: In search for a cure) criteria: abdominal pain + elevated amylase/lipase + imaging findings. Postoperative complications were tracked for a minimum of 12 months.

Data analysis

Descriptive statistics were applied. Categorical variables were presented as frequencies and percentages. Continuous variables were expressed as means. Data analysis was performed using Microsoft Excel (Microsoft Corporation, Redmond, Washington, United States).

## Results

Demographics and presentation

Of the 147 patients included in the study, 103 were female and 43 were male, yielding a female-to-male ratio of 2.4:1. The mean age at presentation was 3.4 months. Most patients (98%) were born at term. The demographic profile of the cohort is presented in Table 2.

**Table 2:** Demographic characteristics of the cohort (N=147)

Demographic Parameter	Value
Sex	
Female	103 (70%)
Male	44 (30%)
Mean Age at Presentation	3.4 months
Gestational Age at Birth	
Term	144 (98%)
Preterm	3 (2%)

The most common presenting symptom was abdominal pain, reported in 85 patients (58%). In younger infants, this often manifested as nonspecific signs such as irritability, inconsolable crying, or abdominal distension rather than verbalized pain. Other symptoms included jaundice (20%), vomiting (7%), and fever (1%). In addition, 13 patients (9%) were diagnosed antenatally via routine fetal ultrasonography, and eight patients (5%) were diagnosed incidentally during imaging for unrelated conditions. The classical triad of abdominal pain, jaundice, and a palpable right upper quadrant mass, a hallmark presentation described in pediatric choledochal cysts, was observed in only six patients (4%) (Table 3).

**Table 3:** Presenting symptoms in choledochal cyst *comprising abdominal pain, jaundice, and a palpable right upper quadrant mass

Presenting Symptom	Frequency (Percentage)
Abdominal pain	85 (58%)
Icterus	30 (20%)
Antenatally detected	13 (9%)
Vomiting	10 (7%)
Incidental detection	8 (5%)
Fever	2 (1%)
Classical Triad*	6 (4%)

LFTs showed abnormal total serum bilirubin (TSB) in 55 patients (35%), with a mean TSB of 5.8 mg/dL (range: 0.1-14.5 mg/dL). Direct serum bilirubin (DSB) was abnormal in 64 patients (43%) with a mean of 2.42 mg/dL (range: 0.01-10.9 mg/dL). Aspartate aminotransferase (AST) and alanine aminotransferase (ALT) were measured in 140 patients (95.2%), with elevated levels noted in 56 (40.0%) and 60 (42.9%) patients, respectively. The mean AST and ALT values were 190 IU/L (range: 16-674 IU/L) and 164 IU/L (range: 6.9-721 IU/L), respectively. Alkaline phosphatase (ALP) was tested in 118 patients (80.3%) and found to be elevated in 39 (33.1%), with a mean value of 749.7 IU/L (range: 109-3129 IU/L). Gamma-glutamyl transpeptidase (GGT) was measured in 48 patients (32.7%), of whom 29 (60.4%) had elevated levels, with a mean of 558.4 U/L (range: 16-1470 U/L).

Ultrasonography was performed in all patients, MRCP in 114 (77%), and ERCP in 37 (25%). The most common indication for ERCP was pancreatitis (21 patients), followed by choledocholithiasis (11 patients), and coexisting choledocholithiasis with cholelithiasis (fvie patients).

Based on Todani’s classification, type I cysts were the most common, occurring in 63.2% (n = 93) of patients. Within type I, subtype Ia was observed in 34.4% (n = 32), subtype Ib in 11.8% (n = 11), and subtype Ic in 53.7% (n = 50). Type IV cysts were seen in 31.3% (n = 46) of patients, of which 93.4% (n = 43) were type IVa and 6.5% (n = 3) were type IVb.Types II and III were not seen in our cohort. APBJ was identified in 21 patients (14%). Among the 14% (n=21) of patients with anomalous pancreaticobiliary junctions, 42.9% (n=9) had associated pancreatitis.

Pancreatitis was documented in 29 patients (20%) and included acute (n=22), recurrent (n=3), chronic calcific pancreatitis (n=2), and cases associated with pancreatic divisum (n=2) (Table 4).

**Table 4:** Types of pancreatitis observed

Type of Pancreatitis	Frequency (Percentage)
Acute pancreatitis (At admission)	16 (55.2%)
Acute pancreatitis (on follow-up)	6 (20.7%)
Recurrent pancreatitis	3 (10.3%)
Chronic calcific pancreatitis	2 (6.9%)
Pancreatic divisum	2 (6.9%)

Associated anomalies included cholelithiasis in 15% and choledocholithiasis in 20%, with both present in 4% of patients. Renal anomalies were seen in 18 patients (12%), gastrointestinal anomalies in 12 (8%), and cardiac anomalies in four (3%) (Table 5).

**Table 5:** Systemic anamolies

System Involved	Abnormality	Frequency (Percentage)
Renal	Pelvic ureteral junction obstruction (n=2) and Hydroureteronephrosis (n=2)	4 (9.4%)
	Polycystic kidney disease	4 (9.4%)
	Increased echogenicity of kidneys	3 (7.1%)
	Medullary Nephrocalcinosis	2 (4.8%)
	Nephrotic syndrome	2 (4.8%)
	Fused Kidney	2 (4.8%)
	Medullary sponge kidney	1 (2.4%)
Gastrointestinal tract	Inguinal hernia	5 (11.8%)
	Biliary atresia	2 (4.8%)
	Duodenal atresia	1 (2.4%)
	Meckel’s diverticulum	1 (2.4%)
	Portal vein thrombosis	1 (2.4%)
	Congenital diaphragmatic hernia	1 (2.4%)
	Sclerosing cholangitis	1 (2.4%)
Cardiac	Tetralogy of Fallot	1 (2.4%)
	Peripheral pulmonary stenosis	1 (2.4%)
	Transposition of great arteries, Ventricular septal defect , pulmonary atresia	1 (2.4%)
	Ventricular septal defect	1 (2.4%)
	Tetralogy of Fallot	1 (2.4%)
Miscelleneous	Hereditary Spherocytosis	2 (4.8%)
	Congenital Hypothyroidism	2 (4.8%)
	Systemic juvenile idiopathic arthritis	1 (2.4%)
	Subarachnoid haemorrhage	1 (2.4%)
	Choroid plexus neoplasm	1 (2.4%)

Of the 147 patients, 110 (75%) underwent surgery. Twenty-three patients (15%) were lost to follow-up, and 14 (9.5%) remained under clinical observation without surgery. The mean age at surgery was 4.1 years, and the mean interval from presentation to surgery was seven months. Roux-en-Y hepaticojejunostomy was the preferred surgical approach.

Histopathological findings showed gallbladder congestion in 72% of patients. Other findings included Rokitansky-Aschoff sinus changes, chronic cholecystitis, metaplastic epithelium (3%), and hyperplastic epithelium (Table 6).

**Table 6:** Histopathology seen with cysts

Histopathology	Frequency (Percentage)
Gall bladder congestion	79 (71.8%)
Gall bladder and Rokitansky-Aschoff sinus congestion	14 (12.7%)
Chronic cholecystitis	7 (6.3%)
Metaplastic changes in epithelium of cyst	4 (3.6%)
Eosinophilic cystitis	2 (1.8%)
Hyperplastic epithelium	2 (1.8%)
Lymph node hyperplasia	2 (1.8%)

Long-term complications included chronic liver disease (CLD) in 11 patients (7%) and portal hypertension in six patients (4%). 

Caroli’s disease

Caroli’s disease was diagnosed in eight patients (5.4%), of whom five were male. Presentations included abdominal pain (n=3), jaundice (n=2), incidental detection (n=1), and screening due to an affected sibling (n=2). Two sets of siblings were identified in this group. Seven of the eight patients had renal anomalies: four had polycystic kidney disease, one had medullary sponge kidney, one had nephrocalcinosis, and one had a fused ectopic kidney. Three patients had inguinal hernia. Genetic testing in one sibling pair revealed an exon 15 variant in the *PKD* gene. Among these eight patients, two developed CLD, two showed altered liver echotexture, and one had portal hypertension. Six patients remain on follow-up, while two were lost to follow-up.

## Discussion

CCs are rare congenital anomalies predominantly seen in the pediatric population. The female predominance observed in our cohort (70%) aligns with international data, which report female-to-male ratios ranging from 3:1 to 4:1 [9,10]. While the exact cause of this sex discrepancy remains unclear, hormonal influences and genetic predispositions have been proposed [11,12]. In our study, the mean age at presentation was 3.4 months, with patients ranging from neonates to adolescents. This is consistent with previous studies indicating that although CCs can present at any age, they are most commonly diagnosed in early childhood [13].

Abdominal pain was the most frequent symptom (58%), followed by jaundice (20%) and vomiting (7%). Antenatal detection occurred in 13 cases (9.0%), consistent with the increasing trend in early diagnosis using fetal ultrasonography as highlighted in Schroeder et al.'s study [14]. However, the classical triad of abdominal pain, jaundice, and a palpable mass was seen in only 4% of patients, reinforcing the often nonspecific nature of CC presentation and the potential for delayed diagnosis.

LFTs were abnormal in many cases. TSB and DSB were elevated in 35% and 43% of patients, respectively. Transaminases, AST and ALT, were also elevated in 40% and 43% of cases, respectively. These findings underscore the importance of comprehensive LFT evaluation in the diagnostic workup of suspected CCs.

Ultrasonography was employed in all cases as the initial imaging modality, with MRCP performed in 77% of patients to confirm the diagnosis and aid surgical planning. ERCP was used in 25% of cases, mainly for patients with coexisting pancreatitis or choledocholithiasis. The most common indication for ERCP was pancreatitis (21 cases), followed by choledocholithiasis (11 cases). These findings highlight ERCP’s dual diagnostic and therapeutic utility in selected CC cases.

In our cohort, type I cysts were most common (63%), followed by type IV (31%), and type V (Caroli disease) in 5.4%. These findings are consistent with global literature, which also notes type I as the most prevalent subtype [15]. Among type I cysts, type Ic was the most frequent. No cases of type II or III cysts were observed. APBJ was identified in 14.3%, and notably, 42.9% of these patients had associated pancreatitis. This supports the well-established role of APBJ in predisposing to pancreatitis and potential malignant transformation due to reflux of pancreatic secretions into the biliary tract [1,13]. The embryological association of APBJ with CCs emphasises the importance of intraoperative assessment and long-term surveillance.

Pancreatitis was seen in 20% of patients, with diverse presentations: acute (n=16), recurrent (n=3), and chronic calcific pancreatitis (n=2). Pancreatic divisum, an anatomical variant associated with impaired drainage and recurrent pancreatitis, was noted in two cases. The strong correlation between CCs and pancreatitis supports early surgical intervention to minimise recurrent inflammation and long-term pancreatic damage.

Cholelithiasis and choledocholithiasis were observed in 15% and 20% of cases, respectively, with both present in 4%. Their co-occurrence further complicates the clinical picture and underscores the need for thorough preoperative imaging.

Surgical intervention remains the definitive treatment for CCs. In our cohort, 110 patients (75%) underwent surgery, with a mean age at surgery of 4.1 years and a mean delay of seven months from diagnosis to procedure. Roux-en-Y hepaticojejunostomy was the preferred approach, offering reliable biliary drainage and reducing anastomotic complications. Histopathological examination showed gallbladder congestion in 72% of specimens. Metaplastic epithelial changes were noted in 3%, suggesting a potential risk of malignant transformation. Other findings included chronic cholecystitis, hyperplastic epithelium, and Rokitansky-Aschoff sinus formation, likely secondary to chronic biliary stasis.

Caroli’s disease, a rare intrahepatic variant of CC, was diagnosed in 5% of the cohort. Notably, two sets of siblings were identified, suggesting a possible genetic basis. In one sibling pair, genetic testing revealed an exon 15 variant in the *PKD* gene, consistent with autosomal recessive polycystic kidney disease. Renal anomalies were found in 87% of patients with Caroli’s disease, further supporting this association. Two patients developed chronic liver disease, and one had portal hypertension, indicating the progressive nature of the condition and the need for long-term hepatobiliary monitoring. These findings warrant further genetic evaluation in suspected familial cases

Despite surgical intervention, long-term complications occurred in a subset of patients. CLD developed in 7%, and portal hypertension in 4%. These findings highlight the necessity of structured postoperative follow-up to monitor for delayed hepatic complications. A total of 23 patients (15%) were lost to follow-up, while 14 (9.5%) remained under observation without surgical treatment. The relatively high loss to follow-up rate underscores the need for better patient education and formalized long-term monitoring protocols.

Our findings are broadly consistent with global literature, reaffirming the predominance of Type I cysts and a female preponderance [13]. Notably, unlike larger adult-centric cohorts such as in the study by Soares et al. [13], where only two pediatric cases had documented APBJ, we observed a substantially higher incidence of APBJ in children, frequently associated with pancreatitis. This underscores the need for vigilant evaluation of pancreaticobiliary junction anomalies even in younger age groups. However, the notably high prevalence of Caroli’s disease in our cohort warrants further genetic and epidemiological exploration. Multicenter studies would help clarify regional variations in CC presentation, genetic predispositions, and long-term outcomes. Furthermore, standardized guidelines for follow-up could mitigate the risk of late complications such as cholangiocarcinoma in patients with residual or recurrent biliary anomalies.

Limitations

The present study has certain limitations. As a retrospective audit conducted at a tertiary care referral centre, the findings may be influenced by selection bias and a higher representation of complex or advanced cases. A proportion of patients (15%) were lost to follow-up, which may have led to an underestimation of delayed or long-term complications. Additionally, gamma-glutamyl transferase (GGT) levels were not uniformly available, thereby limiting the biochemical correlation. These factors may affect the generalisability of the results and underscore the need for larger multicentre prospective studies to validate these findings.

## Conclusions

This study represents one of the largest single-centre analyses of pediatric choledochal cysts in South India, offering valuable insights into their epidemiology, clinical spectrum, and surgical outcomes. Our findings underscore the critical importance of early diagnosis, timely surgical intervention, and structured long-term follow-up in improving patient outcomes and reducing complications. Future research should prioritise the investigation of genetic predispositions, standardised surveillance protocols, and multicenter collaborations to advance the understanding and management of this rare but clinically significant biliary anomaly.
